# A study on anxiety and depression symptoms among menopausal women: a web based cross sectional survey

**DOI:** 10.3389/fpubh.2024.1467731

**Published:** 2024-12-16

**Authors:** Geetha Kandasamy, Dalia Almaghaslah, Mona Almanasef

**Affiliations:** Department of Clinical Pharmacy, College of Pharmacy, King Khalid University, Abha, Saudi Arabia

**Keywords:** menopause, women, anxiety, depression, GAD-7, PHQ-9

## Abstract

**Background:**

An essential part of aging is menopause, which indicates the final phase of the female reproductive cycle. The objective of this research was to assess anxiety and depressive symptoms among menopausal women in Asir region, Saudi Arabia.

**Methods:**

The cross-sectional survey was carried out in February to June 2024, using a random sampling procedure, study participants were selected. All menopausal women including <50 and ≥60 years old, were included, and symptoms of depression and anxiety were collected using Patient Health Questionnaire-9 (PHQ-9) Generalized Anxiety Disorder-7 (GAD-7) respectively.

**Results:**

Of the 396 menopausal women, the majority 170 (42.9%) were <50 years old and 92 (23.2%) were between 50 and 54 years old. Two hundred eighty-one were married (71%) and 273 (68.9%) were literate. Most of them, 229 (57.8%) got married at <18 years and 196 (49.5%) have a parity of 3–5 and 189 (47.7%) women attained menopause at <50 years old. In terms of menopausal symptoms, 268 (67.7%) women experienced hot flushes; 252 (63.6%) had night sweats and were more irritable 256 (64.6%) followed by 244 (61.6%) had decreased sexual desire. In general, 258 (65.2%) and 206 (52.02%) menopausal women reported having depression and anxiety symptoms, respectively. Married (OR = 0.317; 95% CI = 0.182–0.551, *p* = 0.000) and literate (OR = 0.518; 95% CI = 0.309–0.868, *p* = 0.013) are less likely to be in depression compared to widowed/separated individuals. Literates (OR = 0.271; 95% CI = 0.165–0.443, *p* = 0.000), are less likely to have anxiety compared to illiterates.

**Conclusion:**

The study found that a significant percentage of menopausal women had depression and anxiety symptoms. These results emphasize the significance of screening and assessing women experiencing anxiety and depression symptoms throughout the menopausal transition. To alleviate menopausal symptoms, it is also suggested to conduct activities to educate menopausal women, such as a health awareness program in shopping centers and other public places, etc.

## 1 Introduction

An essential part of aging is menopause, which corresponds with the final phase of the female reproductive cycle. It is estimated that 1.2 billion women are expected to reach postmenopause by 2030. Women may be more susceptible to depression and anxiety during periods of hormonal fluctuations, such as puberty, pregnancy, the postpartum phase, and the perimenopause stage. Women were also found to have a higher incidence of both depressive and anxiety disorders than men because of menopause (1–12). During this phase of women life, menopause causes a number of complex changes, including emotional and social alterations (4).

Postmenopausal women, who are increasingly common, represent an important area of focused research. The natural aging process of reproduction is linked to several signs. Mental discomfort is significant because it worsens throughout the menopause transition. As a result of biological aging, the shift from a potentially reproductive to a non-reproductive state typically takes years and is neither instantaneous or abrupt (5, 6). Because there may be a connection between age at natural menopause and the likelihood of developing certain diseases, this is an interesting area of research (4). Studies show that during the menopausal transition, anxiety (7) and depression (8) are more common in women and that anxiety (9) and depression (10) are approximately twice as prevalent in women as in men. Depression is a major worldwide public health concern. Middle-aged women who suffer from depression may experience problems with resilience, sleep, sexual activity, and general quality of life. It also increases the risk of other health problems such as low bone density, osteoporotic fractures, coronary heart disease, metabolic syndrome, and cognitive impairment (11, 12). Recent meta-analyses have confirmed a strong association between increased menopausal age and depression, anxiety, and stress (13). Early detection and treatment of depression are essential to preventing major consequences. Many longitudinal and cross-sectional studies that have looked at depression in women in their midlife have found that the prevalence of depressive symptoms and depression disorders during the menopause phase is higher than those experienced during the premenopausal period, specifically among women who have a history of depression (14).

However, little is known about the prevalence of postmenopausal depression. Little research on anxiety in midlife women, and those conducted have typically examined the connection between stress and vasomotor symptoms as compared with menopausal symptoms. According to the results of Women’s Health Across the Nation (SWAN), a notable community-based, multiethnic, longitudinal study on ovary aging, anxiety is prevalent during the menopause phase of menopause and in the early postmenopausal period (9). Nevertheless, more investigation is needed to comprehend the connection between anxiety and the menopausal stage completely. The objective of this research was to assess anxiety and depressive symptoms among menopausal women in Asir region, Saudi Arabia.

## 2 Methods

### 2.1 Study design, setting and sampling procedure

A web-based cross-sectional survey was carried out from February to June 2024, using a convenience and snowball sampling procedures. Detailed information about the conduct of the study (participant information sheet) was included in the survey cover page along with a consent statement indicating that submitting the questionnaire implies consent to participate in the study. To make sure they could participate willingly, the participants were informed that the questionnaire was anonymous and participation was optional. Furthermore, all data were kept confidential and used only for study. Based on the estimated total population of women in the Aseer region of Saudi Arabia, the sample size was determined using the Raosoft online sample size calculator with a predefined margin of error of 5% and a confidence level of error of 95%. The reliability was increased and the error rates were decreased by choosing the desired sample size of *n* = 384. Of the 433 women who filled the survey, 37 were eliminated because they provided insufficient information.

### 2.2 Inclusion criteria

All menopausal women (perimenopausal phase or postmenopausal phase), including married, single, separated/widowed, <50 and ≥60 years old, were included.

### 2.3 Exclusion criteria

This study excluded menopausal women who had undergone a hysterectomy, took menopause-inducing medicines, woman with thyroid disorders had a history of mental illness or had been diagnosed with it.

### 2.4 Data collection

An online self-administered questionnaire created using Google Forms and advertised through numerous social media channels, including Facebook, Twitter, WhatsApp, and Instagram along with an introductory statement highlighting the inclusion and exclusion criteria. The questionnaire collected the following information: Age in years, literacy status, marital status, age at marriage (if applicable), parity, age at menopause (years), and menopausal symptoms. Symptoms of depression and anxiety was collected using the Patient Health Questionnaire-9 (PHQ-9) and Generalized Anxiety Disorder-7 (GAD-7) questionnaire, respectively.

### 2.5 Patient Health Questionnaire-9

PHQ-9 uses the diagnostic criteria for depression (7) as a basis for its items, which focuses on patient’s experiences during the last 2 weeks. It is intended to collect data on the degree of interest in engaging in activities, feeling sad or depressed, having trouble sleeping, energy levels, eating habits, self-perception, concentration, speed of functioning, and suicidal thoughts. Responses on each survey item ranged from “0” (not at all) to “3” (nearly daily). PHQ-9 scores range from 0 to 27. Severity of the depression can be assessed according to the following scale: 0–4 none, 5–9 mild, 10–14 moderate, 15–19 moderately severe, and 20–27 severe (15). The reliability of PHQ-9 was excellent as indicated by Cronbach’s *α* equals to 0.86 (16).

### 2.6 Generalized Anxiety Disorder-7

GAD-7 is a self-reported questionnaire used for generalized anxiety disorder screening, severity measurement, and assessment (17). There are seven questions in GAD-7. The seven items measure the following: (a) feeling uneasy, tense, or on edge (b) being able to put an end or control worrying (c) worrying excessively about various issues; (d) difficulty relaxing (e) restlessness (f) getting quickly agitated or irritated and (g) feeling terrified that something terrible might occur. For responses “not at all,” “a few days,” “more than half the days,” and “nearly every day,” respectively, scores of 0, 1, 2, and 3 are assigned to determine the GAD-7 score. The final score is calculated by summing the scores of the seven questions. Five, ten, and fifteen were the thresholds for mild, moderate, and severe anxiety, respectively. Ten was used as the cutoff point to divide anxiety and despair into two groups. A score below 10 indicates mild depression or anxiety, whereas a score above 10 indicates moderate depression or anxiety. The anxiety thresholds were as follows: 0–4, 5–9, 10–14, and 15–21, respectively, for no anxiety, mild anxiety, moderate anxiety, and severe anxiety (18). Anxiety and depression were divided into two groups using a cutoff point of 10. A score of <10 was classified as category 1, indicating mild depression or anxiety, while a score of more than 10 indicates moderate to severe depression or anxiety. The reliability of the GAD-7 scale was good as indicated by Cronbach’s *α* of 0.87 (19).

### 2.7 Ethics statement

The study (ECM 2024-211) ethics approval was granted by the King Khalid University Research Ethics Committee. The questionnaire’s cover page now included a consent statement stating that answering the questions indicates your agreement to take part in the study. All data was kept private and utilized exclusively for academic purposes.

### 2.8 Statistical analysis

Data analysis was done using the Statistical Package for the Social Sciences (SPSS) version 20.0. Sociodemographic and other variables were described using descriptive statistics such as percentage and frequencies. The study employed the logistic regression (LR) model to determine the important determinants of anxiety and depression in the menopausal women participated in the study.

## 3 Results

Of the 396 menopausal women, 42.9% were under 50 years old, 23.2% were between 50 and 54 years old, 16.2% were between 55 and 59 years old, and 17.7% were over 60 years old. Among the study population, 31.1% were illiterate and 68.9% were literate. Additionally, 71% were married, and 29% were widowed or separated. Only 42.2% were married at age < 18, whereas 57.8% got married at age 18 or older. Regarding parity, 19.9% had a parity <3, 49.5% had a parity of 3–5, and 30.6% had a parity ≥6. A total of 47.7% of participants were ≥50 years old at menopause, 38.1% were aged 45–49 years, and 14.1% were <45 years old (Table 1).

**Table 1:** Variables of study population.

Variable	Category	Number (%)
Age (years)	<50 years	170 (42.9)
	50–54 years	92 (23.2)
	55–59 years	64 (16.2)
	≥60 years	70 (17.7)
Literacy status	Literate (obtained formal education)	273 (68.9)
	No formal education	123 (31.1)
Marital status	Married	281 (71)
	Widowed/separated	115 (29)
Age at marriage (years)	<18	167 (42.2)
	≥18	229 (57.8)
Parity	<3	79 (19.9)
	3–5	196(49.5)
	≥6	121(30.6)
Age at menopause (years)	<45	56 (14.1)
	45–49	151 (38.1)
	≥50	189 (47.7)

In terms of menopausal symptoms, 67.7% experienced hot flushes; 63.6% had night sweats; 55.6% reported sleep issues; 51.5% had dry or sore vagina; 61.6% experienced decreased sexual desire; 59.6% had aching or painful joints; 47.5% had headaches; 42.7% reported sore breasts; 39.9% had nocturia; 46% felt more irritable; 64.6% reported irritability; and 54.3% experienced memory problems. Details are provided in Table 2.

**Table 2:** Menopausal symptoms of study population.

Menopausal symptoms	Yes number (%)	No number (%)
Hot flushes	268 (67.7)	128 (32.3)
Night sweats	252 (63.6)	144 (36.4)
Sleep problems	220 (55.6)	176 (44.4)
Dry/Sore vagina	204 (51.5)	192 (48.5)
Sexual desire decreased	244 (61.6)	152 (38.4)
Aching/Painful joints	236 (59.6)	160 (40.4)
Dizziness	188 (47.5)	208 (52.5)
Headache	223 (56.3)	173 (43.7)
Sore breast	169 (42.7)	227 (57.3)
Nocturia	158 (39.9)	238 (60.1)
Palpitation	182 (46)	214 (54)
More irritable	256 (64.6)	140 (35.4)
Trouble with memory	215 (54.3)	181 (45.7)

Overall, 65.2% reported depression symptoms and 52.02% reported anxiety symptoms. Among the participants, 13.38% reported no depression; 21.46% reported mild depression; 23.23% reported moderate depression; 32.83% reported moderately severe depression; and 9.09% reported severe depression (Table 3). In terms of marital status, married (OR = 0.317; 95% CI = 0.182–0.551, *p* = 0.000) are less likely to have depression compared to widowed/separated individuals. This difference is statistically significant (*p* < 0.05). Literate (OR = 0.518; 95% CI = 0.309–0.868, *p* = 0.013), are less likely to have depression compared to illiterates, and this difference is statistically significant (*p* < 0.05). That is, among the independent variables, the regression results show that marital status and women’s literacy status have significant effects on depression levels (Table 4).

**Table 3:** Depression and anxiety scores based on the severity of postmenopausal women.

Patient Health Questionnaire-9 (Depression)	Number (%)
1–4: no depression	53 (13.38)
5–9: mild depression	85 (21.46)
10–14: moderate depression	92 (23.23)
15–19: moderately severe depression	130 (32.83)
20–27: severe depression	36 (9.09)
Depression score ≥ 10	258 (965.2)

Generalized Anxiety Disorder-7 (Anxiety)	Number (%)
0–4: no anxiety	75 (18.94)
5–9: mild anxiety	115 (29.04)
10–14: moderate anxiety	144 (36.36)
15–21: severe anxiety	62 (15.66)
Anxiety score ≥ 10	206 (52.02)

**Table 4:** Multiple logistic regression analysis for depression and anxiety symptoms.

		Depression symptoms								Anxiety symptoms							
Variable	Category	Regression coefficient (B)	Std. error	Wald	df	Prob.	OR	95% Confidence interval for OR		Regression coefficient (B)	Std. error	Wald	df	Prob.	OR	95% Confidence interval for OR	
								Lower	Upper							Lower	Upper
Age (years)	<50 years	−0.402	0.355	1.288	1	0.256	0.669	0.334	1.340	−0.099	0.338	0.086	1	0.770	0.906	0.467	1.757
	50–54 years	−0.222	0.376	0.348	1	0.555	0.801	0.384	1.673	0.079	0.358	0.049	1	0.825	1.082	0.537	2.181
	55–59 years	0.223	0.414	0.290	1	0.591	1.249	0.555	2.810	0.485	0.385	1.586	1	0.208	1.624	0.764	3.453
	≥60 years																
Literacy status	Literate	−0.659	0.264	6.230	1	0.013	0.518	0.309	0.868	−1.307	0.251	27.098	1	0.000	0.271	0.165	0.443
	Illiterate																
Marital status	Married	−1.149	0.283	16.531	1	0.000	0.317	0.182	0.551	−0.392	0.244	2.583	1	0.108	0.676	0.419	1.090
	Widowed/separated																
Age at marriage (years)	<18	−0.078	0.231	0.114	1	0.736	0.925	0.589	1.454	−0.176	0.225	0.616	1	0.432	0.838	0.540	1.302
	≥18																
Parity	<3	−0.118	0.329	0.129	1	0.719	0.888	0.466	1.694	−0.116	0.331	0.123	1	0.726	0.890	0.465	1.704
	3–5	0.404	0.267	2.294	1	0.130	1.497	0.888	2.525	0.306	0.258	1.408	1	0.235	1.358	0.819	2.253
	≥6																
Age at menopause (years)	<45	0.413	0.368	1.255	1	0.263	1.511	0.734	3.110	0.369	0.358	1.066	1	0.302	1.447	0.717	2.918
	45–49	−0.038	0.249	0.023	1	0.879	0.963	0.590	1.570	0.239	0.239	1.004	1	0.316	1.270	0.796	2.027
	≥50																

According to the GAD-7 scale, 18.94% reported no anxiety; 29.04% reported mild anxiety; 36.36% reported moderate anxiety; and 15.66% reported severe anxiety (Table 3). Individual responses for each questions were given in Tables 5, 6 for depression (PHQ-9) and anxiety (GAD-7). Literates (OR = 0.271; 95% CI = 0.165–0.443, *p* = 0.000), are less likely to have anxiety compared to illiterates and this difference is statistically significant (*p* < 0.05). The regression results show that women’s literacy status has significant effects on anxiety levels. The following independent variables age, marital status, age at marriage (years), parity, age at menopause (years) have no significant effects on anxiety levels (*p* > 0.05) (Table 4).

**Table 5:** Responses of Generalized Anxiety Disorder (GAD-7) scale among post menopausal women.

Questions	Response	Total
		*n* (%)
1. Feeling nervous, anxious, or on edge	Not at all	81 (20.45)
	Several days	137 (34.60)
	More than half the days	107 (27.02)
	Nearly everyday	71 (17.93)
2. Not being able to stop or control worrying	Not at all	94 (23.74)
	Several days	136 (34.34)
	More than half the days	117 (29.55)
	Nearly everyday	49 (12.37)
3. Worrying too much about different things	Not at all	76 (19.19)
	Several days	134 (33.84)
	More than half the days	114 (28.79)
	Nearly everyday	72 (18.18)
4. Trouble relaxing	Not at all	87 (21.97)
	Several days	165 (41.67)
	More than half the days	104 (26.26)
	Nearly everyday	40 (10.10)
5. Being so restless that it is hard to sit still	Not at all	103 (26.01)
	Several days	130 (32.83)
	More than half the days	101 (25.51)
	Nearly everyday	62 (15.66)
6. Becoming easily annoyed or irritable	Not at all	83 (20.96)
	Several days	146 (36.87)
	More than half the days	107 (27.02)
	Nearly everyday	60(15.15)
7. Feeling afraid, as if something awful might happen	Not at all	98 (24.75)
	Several days	133 (33.59)
	More than half the days	101 (25.51)
	Nearly everyday	64 (16.16)

**Table 6:** Responses of Patient Health Questionnaire (PHQ-9) scale among post menopausal women.

Questions	Response	Total *n* (%)
1. Little interest or pleasure in doing things	Not at all	82 (20.71)
	Several days	139 (35.10)
	More than half the days	124 (31.31)
	Nearly everyday	51 (12.88)
2. Feeling down, depressed, or hopeless	Not at all	78 (19.70)
	Several days	140 (35.35)
	More than half the days	109 (27.53)
	Nearly everyday	69 (17.42)
3. Trouble falling or staying asleep, or sleeping too much	Not at all	64 (16.16)
	Several days	130 (32.83)
	More than half the days	126 (31.82)
	Nearly everyday	76 (19.19)
4. Feeling tired or having little energy	Not at all	57 (14.39)
	Several days	144 (36.36)
	More than half the days	122 (30.81)
	Nearly everyday	73 (18.43)
5. Poor appetite or overeating	Not at all	76 (19.19)
	Several days	127 (32.07)
	More than half the days	133 (33.59)
	Nearly everyday	60 (15.15)
6. Feeling bad about yourself or that you are a failure or have let yourself or your family down	Not at all	109 (27.53)
	Several days	129 (32.58)
	More than half the days	96 (24.24)
	Nearly everyday	62 (15.66)
7. Trouble concentrating on things, such as reading the newspaper or watching television	Not at all	98 (24.75)
	Several days	119 (30.05)
	More than half the days	118 (29.80)
	Nearly everyday	61 (15.40)
8. Moving or speaking so slowly that other people could have noticed. Or the opposite being so fidgety or restless that you have been moving around a lot more than usual	Not at all	94 (23.74)
	Several days	144 (36.36)
	More than half the days	97 (24.49)
	Nearly everyday	61 (15.40)
9. Thoughts that you would be better off dead or of hurting yourself in some way	Not at all	151 (38.13)
	Several days	108 (27.27)
	More than half the days	100 (25.25)
	Nearly everyday	37 (9.34)

## 4 Discussion

Our study aimed to find the depression and anxiety symptoms among menopausal women. Out of 396 menopausal women, the majority reported experiencing depression and anxiety symptoms. The most common menopausal symptoms included hot flushes, night sweats, sleep issues, vaginal dryness, decreased sexual desire, joint pain, headaches, sore breasts, nocturia, irritability, and memory problems. Over half of the participants had severe menopausal symptoms. Similar findings were reported by Ahlawat et al. (20), with hot flushes, sweating, joint pain, depressed mood, and irritability being the most prevalent. According to Sharma’s study (21), joint and muscular discomfort was the most prevalent symptom at 78.42%, whereas vaginal dryness was among the least prevalent at 39.5%. In contrast, our study found a higher prevalence of vaginal dryness at 51.5%, indicating a difference from Sharma’s findings. Additionally, in our study, the mean score was elevated for physical and mental tiredness but lower for vaginal dryness and nocturia. Similar to our findings, another study reported that a significant proportion of women experience menopausal symptoms. Most research participants indicated experiencing anxiety, followed by heart-related issues, joint and muscular discomfort, sleep disturbances, and overall tiredness, both physical and mental. Irritation was also commonly reported. Hot flashes, recognized as the most prevalent symptom, were noted by a considerable number of women. Additionally, mean scores for symptoms like hot flashes, sweating, and joint discomfort showed statistically significant differences (22).

Inversely, less menopausal symptoms were seen in other research (23). This is supported by additional research that indicates women who have hot flashes are more prone than those who do not to suffer from depression (24). Moreover, the available data indicate a clear correlation between high prevalence of anxiety and depressive symptoms in postmenopausal women. These results corroborate findings from a few previously published studies (25, 26). Interestingly, anxiety has been frequently and strongly linked to vasomotor symptoms (VMS) (hot flashes and/or night sweats) in studies around the menopause transition.

Of the menopausal women studied, a notable number reported experiencing varying levels of depression. A significant portion indicated mild depression, while others reported moderate and moderately severe depression. Additionally, some women experienced severe depression. The menopause marks the passage from fertility to old age. At this point, a woman’s ovarian function starts to progressively deteriorate, resulting in menopausal syndrome and, frequently, a host of psychological disorders. Age-related changes in the family and social environment led to an increase in physical strain. Sleeplessness, excessive concern, anxiety, and negative psychological issues are common among menopausal women. This exacerbates the depressive symptoms experienced by menopause syndrome patients (27). Similar to our findings, a study by Polisseni et al. found that 36.8% of women experienced depression during menopause (20, 28). Afshari et al.’s (29) study, which was smaller than the current study, reported that the prevalence of depression in postmenopausal women was 59.8%, with 39.8, and 16% of the women having mild depression and moderate depression, respectively. In contrast to our research, another study discovered that 24.76% of females had clinical depression and 21.9% of females had moderate anxiety scores (30). In a Chandigarh study, Khatak et al. (31) found that 61% of peri and postmenopausal women experienced minimum depression, mild depression (38.5%), significant depression (0.5%), and mild anxiety (0.5%). Furthermore, mild anxiety symptoms were reported by 29.5% of the women and moderate anxiety symptoms by 1% of the women (31).

Based on the GAD-7 scale, many participants reported mild anxiety, with a notable number also experiencing moderate anxiety. Furthermore, some women reported severe or extremely severe anxiety. Similar to our research, another study found that anxiety disorders were common in postmenopausal women and that the severity of the disorder was correlated with the severity of the menopausal syndrome (27). This was consistent with previous study results (32). It is challenging to ascertain if vasomotor symptoms (VMS) are the cause of the observed correlation between anxiety and menopausal stage because VMS are very common during the transition (33, 34).

Our findings are corroborated by Yadollahi et al. (35), who suggest that improving health literacy is associated with changing behavior and actively participating in decision-making. An adequate degree of health literacy and quality of life have already been found to positively correlate among Iranian research participants (36). Our study has a number of limitations because of its self-administered survey and cross-sectional methodology, including the possibility for recall bias during data collection. Moreover, because of the approach of the study, there is limited generalizability of the results obtained.

## 5 Conclusion

The study found that a significant percentage of menopausal women had depression and anxiety. These results emphasize the significance of screening and assessing women experiencing anxiety and depression symptoms throughout the menopausal transition. To alleviate menopausal symptoms, it is also suggested to conduct activities to educate menopausal women, such as a health awareness program in shopping centers and other public places, etc.

## Data Availability

The original contributions presented in the study are included in the article/supplementary material, further inquiries can be directed to the corresponding author.
